# Tracking the Routes of Cancer: The Microevolution of Cancer-Associated Fibroblasts

**DOI:** 10.3390/cancers15215275

**Published:** 2023-11-03

**Authors:** Jarosław Czyż

**Affiliations:** Department of Cell Biology, Faculty of Biochemistry, Biophysics and Biotechnology, Jagiellonian University, Gronostajowa 7, 30-387 Krakow, Poland; jarek.czyz@uj.edu.pl

Cancers are heterogeneous, multicellular societies that constitute solid tumors which comprise the neoplastic progenies of the tumor-initiating cell and the progenies of "un-transformed" tumor-infiltrating cells. Heterogeneous genomic and epigenetic instability, sustained proliferative signaling, resistance to growth-limiting and pro-apoptotic signals, replicative immortality, metabolic plasticity, and invasive potential underlie the phenotypic diversification of neoplastic cells [1]. The populations of stromal, endothelial, and immune cells add to the dynamics of this diversity via the contribution to the nutritional supply routes, regulatory paracrine loops, and structural scaffolds. Consequently, the coordinated expansion and extinction of discrete transformed (neoplastic) and untransformed cell lineages prompt the microevolutionary processes that underlie tumor growth, adaptation, and systemic dissemination (metastasis) (Shlyakhtina, Y. et al., 2021). Among them, cancer-associated fibroblasts (CAFs) have been recognized as the heterogeneous category of non-transformed tumor cells, which is crucial for cancer microevolution [2]. 

Originally, CAFs were categorized as the sentinels of mechanical scaffolds, which contributed to the regionalized deposition and remodeling of extracellular matrices in the tumors. However, we have recently witnessed several major breakthroughs in the field of CAF biology that point to the complexity of their impact on cancer homeostasis [3]. The progress in single-cell profiling techniques [4,5] identified CAFs as heterogeneous signaling "hubs", which integrate paracrine, biomechanical, and juxtacrine communication systems with the structural homeostasis of solid tumors. This notion has been elegantly addressed in the recent paper of Sebastian et al., who demonstrated the coexistence of mechanoactive (αSMA^high^), inflammatory, and MHC class II-expressing CAF lineages in breast cancer. The corresponding co-existence of desmoplastic, contractile, aggressive (inflammatory), and immune CAF lineages, and the progressive expansion of tumor-supporting CAFs, was observed in the tumors originating from other tissues [6]. Conceivably, the cooperation of heterogeneous CAF lineages underlies the contribution of tumor stroma to cancer promotion, progression, and stress resistance. In turn, their selective expansion and extinction account for the microevolution of CAFs and their synchronization with the microevolution of tumors.

The tumor-supportive effects of CAFs are largely executed via their effects on the quality and quantity of the extracellular matrix (ECM). The ECM constitutes the physical scaffold of the tumors, which mediates the intercellular transmission of mechanical forces. It also coordinates local paracrine signaling pathways via the accumulation or sequestration of growth factors and cytokines. The ECM-mediated spatiotemporal integration of the paracrine, biomechanical, and juxtacrine systems of intercellular communication governs tumor homeostasis [7]. The interrelations between intratumoral ECM dynamics and the heterogeneity of phenotypic CAF signatures have recently been reviewed by Belhabib et al. They summarized the consequences of the heterogeneous contractile and secretory activities of CAFs for the abundance, chemical composition, and mechanical properties of the ECM. Next, CAF-dependent ECM dynamics were discussed in terms of the formation of invasive front, metastatic cascade, angiogenesis, immune cell infiltration, and the drug-resistance of tumors. The complexity of the interrelations between cancer microevolution and phenotypic CAF signatures is exemplified by the subtle roles of desmoplastic and contractile CAFs in the regulation of cancer invasion. The extensive desmoplasia impedes tumor growth and progression through abundant collagen deposition. However, ECM composition can change towards the formation of invasive-supporting microstructures via CAF-induced mechanical or enzymatic realignment. Subtle shifts in the cooperative activity of ECM-producing CAFs and their highly contractile (αSMA^high^) counterparts determine the local thickness and alignment of collagen fibers to augment the invasion of adjacent neoplastic cells (via the formation of motility routes) or to attenuate this process (via the constitution of the local tissue barriers). Notably, the interactions between the neoplastic cells and CAFs are reciprocal, i.e., the microevolution of a CAF phenotype can be affected by mechanochemical signals from neoplastic cells. Moreover, “pro-invasive/inflammatory” CAFs can modulate these feedback loops via the establishment of auxiliary paracrine loops involving the gradients of cytokines, growth factors (TGF-β), and metalloproteinases. These facts illustrate the dynamics of the CAFs’ contribution to the communication networks that coordinate tumor development and progression. The identification (i) of the consequences of CAF selective expansion/microevolution for the quality of these networks and for the natural history of cancer, (ii) of the phenotypic traits of CAFs that determine their pro-invasive activity, and (iii) of the selection mechanisms that account for a selective expansion of discrete CAF lineages, represents the real challenge for current oncology.

The long-term consequences of the misbalanced activity of “desmoplastic” and “inflammatory” CAFs for tumor microevolution have been illustrated by the recent study of Kang et al. They distinguished two phenotypically stable CAF sub-types in the head and neck squamous cell carcinomas (HNSCCs). CAFs isolated from benign tumors (CAF-D), which were characterized by well-demarcated boundaries between the ECM and neoplastic cells, did not enhance the invasion of HNSCC cells in vitro. In turn, the absence of a such boundaries in aggressive HNSCC tumors correlated with the invasion-promoting (CAF-P) phenotype of the residential CAFs. Differences in the phenotypic signatures of CAF-D and CAF-P lineages did not correlate with their αSMA expression pattern, which shows the versatility of αSMA^positive^ CAF lineages. Instead, transcriptomic assays revealed relatively high levels of collagen (COL3A1 and COL6A6) encoding transcripts in CAF-D cells. The quality of CAF-P transcriptome and secretome (incl. relative abundance of CXCL12 and metalloproteinases, respectively) and pro-invasive CAF-P effects in the HNSCC spheroid model suggest the paracrine mechanism of their activity. In turn, the pro-invasive effect of COL3A1 and COL6A6 knock-down in CAF-D cells indicates a certain level of antagonism between the ECM-depositing and pro-invasive activities of CAFs. This study illustrates how the reciprocal reprogramming of tumor cells and the cancer microenvironment contribute to the diverse patterns of cancer microevolution. The considerable phenotypic stability of CAF-D and CAF-P populations in vitro may additionally petrify these benign and pro-invasive patterns in vivo. 

In another study on the determinants of pro-invasive CAF activity, Czekay et al. reviewed exemplary phenotypic CAF signatures that are potentially crucial for pro-invasive intratumoral communication networks. For instance, α5 integrin^high^ CAFs apparently coordinate the early steps of metastatic cascade in ovarian cancer. In turn, the plasminogen activator inhibitor (PAI)-1/SERPIN appears as a prominent regulator of pro-invasive CAF-mediated ECM remodeling. Depending on the cellular context, PAI-1 can mediate the pericellular ECM degradation and/or coordinate the local adhesive and invasive cell properties. The significance of these activities for the microevolution of tumor invasiveness is illustrated by the postulated central role of myofibroblastoid (αSMA^high^/PAI-1^high^) CAFs in the feedback loops that integrate CAFs, mesothelium, and adjacent neoplastic cells with the local immune systems, including cancer-associated macrophages. Their paracrine cooperation can further promote the vascularization, invasive front formation, and drug resistance in versatile cancer systems. Collectively, the correlation between PAI-1 levels and reduced patient survival indicates that the selective expansion of PAI-1^high^ CAFs contributes to the evolution of the pro-invasive tumor microenvironment. Subtle interactions between the discrete CAF lineages synchronize their phenotypic evolution with the microevolution of whole tumor societies. An open question concerns the selective factors and cell adaptation processes that synchronize the microevolution of tumors and their residential CAF lineages. Apparently, local microenvironmental deficits, such as hypoxia and chemotherapeutic stress, affect the quality and direction of local CAF microevolution. This is illustrated through the signs of PAI-1^high^ CAF activation in response to these cues [5]. 

The consequences of local niche deficits for CAF activation and microevolution have been more extensively reviewed in the recent paper by Kim et al. They provided an elegant summary of the hypoxia in solid tumors, its consequences for the heterogeneity of phenotypic CAF signatures, and the consequences of the local CAF reactivity to hypoxia for tumor homeostasis and invasiveness. Spontaneous vascular collapses can induce local nutritional shortages that selectively affect the pro-fibrotic and pro-inflammatory CAF populations in the hypoxic loci with their diverse epigenetic memory, metabolic plasticity and metabolic cooperation potentials (see also Sanford-Crane, H., et al. 2019). They can further rearrange the local feedback loops between CAFs and immune and neoplastic cells to promote the alternative scenarios of anti-invasive fibrosis or the pro-invasive activation of immune and neoplastic cells. Alternative (pro-fibrotic and pro-invasive) scenarios of local tumor microevolution are also triggered by the structural collapses following the ablation of pro-fibrotic CAFs [3,5]. Presumably, the augmented drug-resistance of “pro-invasive” CAFs can promote their local selective expansion and the microevolution of local microenvironments towards the pro-invasive ECM remodeling scenario. In conjunction with the attenuated bioavailability of the drugs in collapsed regions, these events underlie the adverse effects of the CAF ablation regimens of cancer treatment. Thus, the differential sensitivity of pro-fibrotic and pro-invasive CAF lineages to paracrine loops, hypoxia, and/or a “chemotherapeutic tsunami” can redirect the synchronous CAF/cancer microevolution and the natural history of cancer towards the alternative benign or malignant scenarios. The identification of the mechanisms that determine the direction of this process is necessary for the elaboration of new CAF-targeted cancer treatment regimens (Louault, K., et al. 2020, [8]).

Collectively, the microevolution of discrete CAF lineages appears as a prerequisite for cancer promotion and progression. However, further efforts are necessary to elucidate (i) how the microevolution of CAFs is synchronized with the microevolution of the whole tumor, (ii) what the impact of this synchronicity on cancer progression is, and (iii) whether this process can be pharmacologically targeted. At the systemic level, phenotypically discrete CAF lineages of different origins (local mesenchyma, endothelia, epithelia, bone marrow, pericytes, or fibrocytes) and phenotypes (contractility, ECM, and pro-inflammatory secretome) are differentially recruited to the 1^ary^ tumors and their 2^ndary^ descendants. Single-cell studies on the local phenotypic signature of CAFs should help to identify the universal patterns of their impact on the natural history of cancer. At the microenvironmental level, the ECM stabilizing, aligning, and remodeling activity of discrete CAF lineages cooperates with the local paracrine, biomechanical, and juxtracrine feedback loops between CAFs and immune and neoplastic cells in determining the patterns of pro-invasive cancer microevolution. The analyses of the spatiotemporal evolution of this cooperation are necessary to elucidate how the synchronized microevolution of CAFs contributes to the local pro-invasive cancer microevolution. At the single cell level, the sensitivity and reactivity of CAFs to local nutritional deficits (hypoxia) and chemotherapeutic stress is determined by the interrelations between their metabolic plasticity and the activity of drug resistance systems. The identification of these links should help to identify the mechanisms standing behind the disappointing results of the currently tested CAF ablation strategies of cancer treatment. In any case, the appreciation of the significance of phenotypic CAF microevolution and of its synchronicity with the microevolution of tumor ecologies may revolutionize our view on cancer development and treatment. 
